# Unstable hours, unequal health: Gendered occupational health costs of work-hour volatility in the U.S.

**DOI:** 10.1016/j.ssmph.2026.101949

**Published:** 2026-07-13

**Authors:** Sinyee Qianyi Lu, Tim F. Liao

**Affiliations:** University of Illinois Urbana-Champaign, Urbana, IL, USA

**Keywords:** Work-hour volatility, Self-rated health, Precarious employment, Healthy worker effect, Occupational health

## Abstract

**Introduction:**

Work-hour instability is a key feature of precarious employment, yet its population-level health implications remain insufficiently understood and may be obscured by employment-based selection.

**Methods:**

Using the Current Population Survey linked across two four-month observation periods separated by an eight-month unobserved interval, we construct a work-hour volatility index from within-person changes in work hours and estimate gender-stratified multinomial logit models to assess whether volatility predicts subsequent transitions in self-rated health (SRH) and health-related work limitation (HRWL) as an additional outcome.

**Results:**

Results show gender-differentiated patterns. Among men, work-hour volatility is associated with a higher likelihood of SRH deterioration while remaining employed. Compared with stable 40-hour weeks across four monthly observations, a volatile pattern of 20-hour weeks in month 1, 60-hour weeks in month 2, 60-hour weeks in month 3, and 20-hour weeks in month 4 was associated with a higher predicted probability of SRH deterioration (38.0% vs. 28.1%). Among women, volatility is not clearly associated with SRH changes; however, under the same contrast, predicted HRWL probability is higher under high volatility than stable hours (5.2% vs. 2.5%).

**Conclusion:**

Our findings suggest that work-hour volatility is a meaningful, gendered dimension of occupational health risk, with implications for both health inequality and labor-force attachment. Policies promoting stable working hours should be considered because they may have public health benefits.

## Highlights

• Work-hour volatility is a meaningful dimension of occupational health risk.
• Negative association between work-hour volatility and subjective health is gender-specific.

## 1 Introduction

For decades, occupational health frameworks focused on time-related risks such as excessive work hours and nonstandard schedules (Erren et al., 2019; Fevang, Fidjeland, Hauge, & Lillebø, 2025; Kivimäki et al., 2015; Sato et al., 2020), aligning with the logic of fundamental cause theory and emphasizing social conditions such as socioeconomic status and social support, rather than proximal factors, as the fundamental causes of diseases (Benach et al., 2014; Frank et al., 2023; Link & Phelan, 1995).

Yet the labor market has changed substantially, and the emerging landscape of precarious work-time arrangements—including long, short, very short, and highly variable hours—presents new risks that traditional frameworks often overlook (World Health Organization & International Labour Organization, 2021; ILO, 2023). U.S. employers increasingly use “just-in-time” scheduling strategies,^1^ which prioritize flexibility for firms over stability for workers (Appelbaum et al., 2003; Choper et al., 2022; Costa, 2003; Lambert et al., 2014, 2019). It undermines workers’ control over their time (Gerstel & Clawson, 2018; Golden and Kim, 2017), erodes work–life balance (ILO, 2023), and contributes to health harms, including psychological distress, sleep disturbances, functional limitations, and even mortality in later life (Donnelly, 2022; Erhel et al., 2024; Harknett et al., 2020; Mai et al., 2019; Schneider & Harknett, 2019). It also generates income inequality, material hardship, and poverty risk (Cai, 2024a, 2024b; Cai & Peck, 2022; Lu & Leicht, 2025), suggesting that work-hour instability is not just inconvenient but structurally consequential. Given these transformations of work, occupational health frameworks must evolve to fully address the temporal dimension of precarity (ILO, 2023).

Several key research gaps remain. First, most evidence emphasizes concrete scheduling practices, leaving open whether more general instability in work hours predicts subsequent health change under population-level inference. Second, the gender patterning of health consequences remains underexplored. Because gender stratifies both exposure to precarious work and responses to health strain—women are more likely to interrupt, reduce, or suspend work after health problems, whereas men more often remain employed as health deteriorates (Boden & Galizzi, 2003; Campos-Serna et al., 2013; Macpherson et al., 2019; Timp et al., 2024)—work-hour instability may surface through different health trajectories by gender. Third, we know little about how these pathways intersect with the healthy worker survivor effect, which may confound the relationship, despite cohort evidence on gendered survivor selection (Arrighi & Hertz-Picciotto, 1994; Buckley et al., 2015; Naimi et al., 2013).

This study presents an original examination of the population-level association between work-hour instability and subsequent health transition with the Current Population Survey (CPS), addressing those gaps in several ways. By treating health-related work limitation as an outcome rather than sample attrition or censoring, the study follows individuals even when deteriorating health leads them to reduce or exit paid employment, instead of removing those cases from analysis. Gender-stratified models confirm the sharp gender differences in health pathways: work-hour instability, or “volatility,” is more likely to be reflected in on-the-job health deterioration among men, and in health-related withdrawal from active employment among women.

## 2 Background

### 2.1 Theoretical gap: precarious work time as a social determinant of health

Consistent with fundamental cause theory, work and occupations shape access to resources such as knowledge, money, power, prestige, and social connections, all of which influence both exposure to health risks and the ability to avoid or mitigate their consequences (Link & Phelan, 1995). Among all relevant factors, precarious work has been increasingly recognized as a critical social determinant of health, particularly in higher-income countries (Benach et al., 2014; Frank et al., 2023). While definitions vary, it typically refers to jobs that are unstable, insecure, and unfavorable in terms of employment relationships, working conditions, employee autonomy, economic and non-economic compensation, access to legal and social protections, and opportunities for career advancement (ILO, 2012; Kalleberg, 2016; Ravenelle & Kowalski, 2022, 2023; Vosko, 2006). Empirically, various forms of precariousness have been linked to poorer self-rated health, lower work satisfaction, increased depressive symptoms, higher occupational injury risk, and even elevated mortality from alcohol- and tobacco-related causes (Benach et al., 2014; Haile, 2023; Quinlan & Bohle, 2009; Virtanen et al., 2003). However, much of this literature has concentrated on the employment relationship itself (Artazcoz et al., 2005; De Cuyper et al., 2008; Virtanen et al., 2004) while overlooking other critical dimensions of precariousness, including unstable or unpredictable work time.

This gap motivates explicitly incorporating precarious work time into research on health disparities, in line with the fundamental cause theory. Colen and colleagues (2024) argue that time—including time at work—is a fundamental yet underexamined resource shaping how individuals navigate risk and protection, with implications for health and survival. Time thus warrants distinct consideration as a social determinant of health; inequalities in access to and control over time—whether for paid labor, healthcare, domestic work, caregiving, or personal time—can directly contribute to health disparities. Consistent with this view, Schneider and Harknett (2019) emphasize that the temporal dimension of precarious work—unstable, unpredictable schedules—is at least as important as the economic dimension in shaping workers’ health and well-being, and it deserves far greater academic attention, though the empirical indicators used to capture such instability may reflect heterogeneous sources, including both employer-driven scheduling practices and worker responses.

Emerging evidence links unstable or unpredictable schedules to adverse outcomes. Routine schedule instability—including on-call shifts^2^, short-notice schedule changes or cancellations^3^, and “clopening” shifts^4^—is associated with elevated psychological distress, poorer sleep quality, and greater morning fatigue (Harknett et al., 2020). Weekend shifts and short-notice changes increase difficulty arranging childcare and intensify parenting stress (Luhr et al., 2022; Usdansky & Wolf, 2008). Time-related instabilities carry serious economic consequences, including income volatility (Lu & Leicht, 2025; Thomas et al., 2022), child poverty (Cai, 2024a), and material hardships such as food insecurity and the unaffordability of basic medical care (Schneider & Harknett, 2021)—all of which carry long-term health risks. Mid-life exposure to worktime precarity can have cumulative effects, contributing to chronic health conditions, functional limitations, and overall poorer health in later life (Donnelly, 2022).

Despite this progress, existing literature typically focuses on particular sectors (retail and food service) and concrete scheduling practices, limiting generalizability. Consequently, we still lack evidence on how work-time instability as a general, population-wide phenomenon—beyond specific scheduling practices like on-call shifts and short-notice shift changes—affects health in the overall labor force.

### 2.2 Gendered pathways: differential observability of health harm

Gender is not a “control variable” in occupational health; it organizes how health is measured, how risks are distributed, and how health problems translate into labor-market consequences. First, self-rated health (SRH) is a socially patterned measure: gendered norms, expectations, and lived experiences shape how symptoms are perceived and reported, and the meaning and predictive validity of SRH can vary across social groups (Read & Gorman, 2010; Woo & Zajacova, 2017). Second, population health itself is gendered—women and men differ in morbidity, disability, and longevity, reflecting gendered exposures and resources across the life course (Krieger, 2003; Short & Zacher, 2022). Third, gender stratifies exposure to occupational risks. Women are more concentrated in service or shift-based roles with ergonomic load, irregular hours, and circadian disruption—conditions linked to sleep problems, cardiometabolic stress, and cumulative physiological wear (Campos-Serna et al., 2013). The sex/gender differences in occupational hazard exposures and in work injury/disability risk are systematic and partly rooted in gendered job assignment and unequal working conditions (Biswas et al., 2021, 2022; Campos-Serna et al., 2013).

Moreover, labor-market responses to health strain are also gender-differentiated. Following occupational injury or illness, women and men often face different constraints and options for maintaining employment, and they incur different downstream consequences. Gender differences are observable in the economic costs of work injury (Boden & Galizzi, 2003), in the duration of work-disability spells (Macpherson et al., 2019), and in long-term sickness absence patterns (Timp et al., 2024). Within a cohort of synthetic vitreous fiber workers, women in poorer health were more likely than men to leave high-exposure jobs or reduce exposure earlier, leaving a healthier subset of women who remain and thereby muting risk estimates for women (Lea et al., 1999). Similarly, in a large Dutch registry of long-term sickness-absence spells, women had longer average absence durations and slower early return-to-work trajectories than men across nearly all diagnostic groups—especially mental and musculoskeletal conditions—highlighting gender-differentiated patterns in the duration of work interruption following health problems (Timp et al., 2024).

Together, these patterns imply two distinct pathways through which adverse working conditions (e.g., precarious work time) may affect health by gender. These gender differences matter not only substantively but also methodologically, because they shape who remains observed in employed-only samples and therefore what “health effects” are even detectable. This motivates the next section, where further methodological challenges are outlined.

### 2.3 Methodological challenges: healthy worker survivor effect

Although hazardous working conditions can harm health, estimating those harms is complicated by the healthy worker survivor effect (HWSE). Over time, workers whose health deteriorates are more likely to reduce exposure—by cutting hours, changing jobs, or exiting the labor force completely—while those who remain are positively selected on health (Lea et al., 1999). This produces a feedback structure in which prior exposure affects subsequent health, health affects subsequent employment and exposure, and employment becomes a time-varying confounder affected by prior exposure—conditions under which standard regression adjustment can fail (Buckley et al., 2015; Naimi et al., 2013). Consequently, models that condition on current employment or restrict analysis to those still observed may yield attenuated estimates of occupational harm (Chevrier et al., 2012).

In response to the HWSE, this study adopts a selection-aware sample and outcome construction that reduces employment-based truncation and partially mitigates the selection bias. Rather than censoring respondents when deteriorating health leads to reduced work, suspended work, or labor-force exit, the analysis treats health-related work limitation as an adverse health transition and follows all respondents from the baseline interview regardless of subsequent employment status in our expanded models. Making health-driven employment change observable—rather than treating it as attrition—approximates less truncated follow-up and shifts attention from continuously employed “survivors” to a broader population at risk. While this strategy does not eliminate selection bias or identify counterfactual causal effects, it reduces the mechanical censoring of employed-only designs and allows gender-differentiated pathways to be observed both within employment and through health-related withdrawal.

This literature therefore motivates two research questions. First, is work-hour instability, or “volatility,” associated with subsequent health outcomes among U.S. workers, and if so, are these associations different for women and men? Second, does the volatility-health association depend on the overall level of weekly work hours? This second question is consistent with the view that work time is multidimensional: workers’ health may be shaped not only by the stability of hours, but also by the amount and organization of time spent at work (Colen et al., 2024; ILO, 2023; Sato et al., 2020; Schneider & Harknett, 2019). Accordingly, the health implications of unstable hours may differ depending on whether volatility occurs within relatively short, standard, or long-hour work arrangements. We therefore examine not only whether work-hour volatility is associated with health, but also whether this association varies by gender and by the level of weekly hours worked.

## 3 Data & methods

This study uses the Current Population Survey (CPS) Annual Social and Economic Supplement (ASEC) linked to the CPS Basic Monthly files, from the Integrated Public Use Microdata Series (IPUMS), provided by the Institute for Social Research and Data Innovation at the University of Minnesota (Flood et al., 2023). Within the CPS, the ASEC is the only supplement fielded annually that includes a self-rated health measure, making it essential for constructing between-year health transitions. We selected data from 2016 to 2024, while excluding 2018-2019^5^ and 2019-2020^6^. Using the CPS 4–8–4 sampling rotation and IPUMS longitudinal identifiers, we link individuals interviewed in ASEC at time *t* ("Year 1") to the same individuals in the same calendar month at *t*+1 ("Year 2"), yielding within-person health trajectories across two consecutive calendar years, with two four-month observation periods separated by an eight-month unobserved interval.

Analytic samples are restricted to working-age (18–64), noninstitutionalized civilians with validated consecutive ASEC interviews, complete linkage across both four-month in-sample spells, and ≥6 Basic Monthly interviews (≥3 per spell) with valid work hours. We then apply the following restrictions based on respondents’ characteristics in Year 1, excluding those with zero/missing hours, full-time students, active-duty military, unpaid family workers, those working part-time for health reasons, and cases with missing analysis variables. These criteria yield a basic employed sample (*N* = 55,062); adding respondents with Year-2 health-related work limitation expands the sample to *N* = 61,131. Additional details are provided in Appendix A. Because the CPS excludes institutionalized populations and relies on self-reported measures, the resulting estimates apply to the civilian, noninstitutionalized population and may not generalize to the institutionalized populations.

The independent variable, work-hour volatility, captures within-person month-to-month instability^7^ in usual weekly hours during the baseline year (ASEC Year 1), reflecting realized variation in work time rather than its underlying sources (e.g., employer-initiated changes, worker preferences, or cyclical demand). Following Cai's approach (Cai, 2024a, 2024b; Cai & Mattingly, 2025; Cai & Peck, 2022), we compute volatility as the standard deviation^8^ of arc changes^9^ in hours across adjacent CPS Basic Monthly interviews^10^:

$$\text{Arc}\;\text{Change}\;\Delta_{i,m}=\frac{{Workhours}_{i,m}-{Workhours}_{i,m-1}}{\left({Workhours}_{i,m}+{Workhours}_{i,m-1}\right)/2\;}$$

$$\text{Volatility}\;\text{Index}\;v_{i}=\sqrt{var\left(\Delta_{i,m}\right)}\text{,}$$

where ${Workhours}_{i,m}$ refers to the usual weekly work hours of individual *i* in month *m*, and ${Workhours}_{i,m-1}$ refers to the same individual's work hours in the preceding month. The numerator of arc change therefore captures the change in hours between two adjacent months, while the denominator scales that change by the midpoint of the two monthly values. This approach therefore yields a symmetric percent change and reduces sensitivity to hour levels. Consequently, $v_{i}=0$ indicates complete stability, while $v_{i}=1$ indicates that the within-person across-time standard deviation of adjacent-month arc changes equals 1. To aid interpretation, Table 1 further presents hypothetical four-month work-hour sequences as illustrative examples of selected volatility values. Summary statistics of this variable as well as all other variables in our analytical samples are provided in Appendix B.

**Table 1:** Hypothetical four-month work-hour sequences producing different volatility values.

Example ID	Hypothetical Work-hour sequence, Months 1–4	Between-month hour changes, (${Workhours}_{i,m}-{Workhours}_{i,m-1})$	Arc changes, $\Delta_{i,m}$	Volatility, $\sqrt{var\left(\Delta_{i,m}\right)}$^a^
1	40, 40, 40, 40	0, 0, 0	0, 0, 0	$v_{i}=0$
2	25, 25, 25, 25	0, 0, 0	0, 0, 0	$v_{i}=0$
3	30, 50, 50, 30	+20, 0, −20	+.50, 0, −.50	$v_{i}=0.5$
4	50, 30, 30, 50	−20, 0, +20	−.50, 0, +.50	$v_{i}=0.5$
5	20, 60, 60, 20	+40, 0, −40	+1.00, 0, −1.00	$v_{i}=1$
6	18, 6, 6, 18	−12, 0, +12	−1.00, 0, +1.00	$v_{i}=1$
7	10, 70, 70, 10	+60, 0, −60	+1.50, 0, −1.50	$v_{i}=1.5$
8	28, 4, 4, 28	−24, 0, +24	−1.50, 0, +1.50	$v_{i}=1.5$

Notes: These examples are hypothetical and use four monthly observations, which generate three adjacent-month changes. Raw hour changes, arc changes, and volatility index calculations are based on the formulas listed above.

a The full equation can be written as: $v_{i}=\sqrt{var\left(\Delta_{i}\right)}=\sqrt{\frac{1}{K_{i}-1}\sum_{m=2}^{M_{i}}{\left(\Delta_{i,m}-\bar{\Delta_{i}}\right)}^{2}}$, where $M_{i}$ denotes the number of months when individual *i* is observed in the data in Year 1; $\Delta_{i,m}$ denote the arc change for individual *i* in month *m*, specifically; $\bar{\Delta_{i}}$ denotes the within-person across-time average arc change for individual i in Year 1.

The dependent variable is derived from self-rated health (SRH). SRH has been widely used as an integrated assessment of physical symptoms and diagnosed conditions, functional limitations, and mental well-being, drawing on information that may not be fully captured by single-domain indicators (Benyamini, 2011; Idler & Benyamini, 1997; Jylhä, 2009). Empirically, SRH has been shown to predict subsequent mortality independent of diagnosed conditions and clinical risk factors, and to correlate with objective indicators such as chronic disease burden, functional limitations, disability progression, and pain-related impairment (DeSalvo et al., 2006; Nielsen et al., 2009; Vogelsang, 2014). In the context of short-run labor-market change, SRH is especially appropriate because early health deterioration often presents first as fatigue, stress, sleep disturbance, or reduced day-to-day functioning—changes that may not yet generate a formal diagnosis but can still meaningfully affect work capacity, attendance, and the ability to sustain employment over the next year (DeSalvo et al., 2006; Jylhä, 2009). Consistent with this logic, research on working conditions routinely employs self-rated or closely related self-reported health outcomes to assess the health consequences of job characteristics (Artazcoz et al., 2005; Burgard et al., 2009; Donnelly, 2022; Erhel et al., 2024; Haile, 2023).

Accordingly, the dependent variable in our baseline models is defined as the between-year change in SRH: change is coded into three categories—improved, stable, or deteriorated—based on movement between Year 1 and Year 2 across the five SRH levels. For instance, a respondent is coded as deteriorated if Year-2 SRH is worse than Year-1 SRH (e.g., excellent to good or fair). In contrast, a respondent is coded as improved if Year-2 SRH is better than Year-1 SRH (e.g., fair to good or very good). If there is no movement across the five SRH categories between years, the respondent will be classified into the “stable” group. This approach follows prior transition-based methods (e.g., Diehr & Patrick, 2001; Sacker et al., 2007).

The expanded dependent variable retains the basic three-category SRH transition outcome —improved, stable, and deteriorated— and adds health-related work limitation (HRWL) as a fourth category, incorporating health-related reductions in or exits from employment that would otherwise appear as attrition in employed-only designs. HRWL captures transitions into reduced, suspended, or exited employment due to health, reflecting both underlying health conditions and the institutional and labor-market contexts shaping responses to those conditions, thereby reducing bias from health-driven employment exit suggested by prior literature (e.g., Boden & Galizzi, 2003). Specifically, HRWL is coded when respondents report any of the following in Year 2: working part-time for health reasons^11^, being absent from work due to illness or medical problems^12^, or being out of the labor force because of illness or disability^13^. Detailed survey items are presented in Appendix A. Respondents who do not meet any of these criteria remain classified under the original three-category SRH transition outcome: improved, stable, or deteriorated.

We model one-year health change from ASEC Year 1 to Year 2 using sex-stratified multinomial logit models (“multinomial transition models”), treating health change as movement between mutually exclusive states observed at two time points, appropriate for a one-step transition window (Breen & Jonsson, 2000; Diehr & Patrick, 2001). Because sex/gender structures SRH reporting, exposure to work conditions, and labor-market responses to health strain, models are estimated separately for women and men^14^ using CPS-reported biological sex and interpreted as gendered processes of exposure, vulnerability, and selection (Boden & Galizzi, 2003; Krieger, 2003; Read & Gorman, 2010).

All models control for baseline five-category SRH, use stable SRH as the reference outcome, and apply ASEC Year-2 person weights (asecwt_2) to represent the Year-2 employed population. Baseline SRH, measured in ASEC Year 1, is the key confounder because the linked CPS-ASEC design allows adjustment for pre-existing health prior to both the baseline volatility window and the Year-2 health transition outcome. Following prior research using similar volatility measures (Cai, 2024a, 2024b; Cai & Mattingly, 2025; Cai & Peck, 2022), we also control for the level of weekly work hours and include an interaction term between work hours and volatility to capture the possibility that the effects of work-hour instability may vary depending on the overall amount of work. All other covariates are likewise measured in Year 1 (on the ASEC month) and include age, marital status, race/ethnicity, educational attainment, number of children and age of the youngest child in the household, and major industry and occupation^15^. Because the main models are stratified by gender, gender is not entered as a covariate. We also include period indicators to account for pandemic-related macro shocks, distinguishing pre-pandemic (2016–2017, 2017–2018), pandemic (2020–2021, 2021–2022), and post-pandemic (2022–2023, 2023–2024) intervals and treating these as broad contextual controls rather than modeling potentially distinct volatility processes across labor-market regimes. This temporal ordering reduces, but does not eliminate, concerns about reverse causation and supports interpretation of the estimates as associations conditional on prior health and observed characteristics rather than as causal effects. Later regression tables report regression coefficients, while the text presents relative risk ratios (RRRs) for the focal independent variable and selected key covariates.

Below, we present the specific formulations of the baseline and expanded multinomial transition models.

**Baseline model (employed-only sample; *N* = 55,062).** The baseline specification estimates three-category SRH transitions—deteriorated (−1), stable (0), improved (+1)—among respondents continuously employed through Year 2. Thus, the model captures health transitions conditional on remaining employed, consistent with conventional designs where employment exit truncates observation (Arrighi & Hertz-Picciotto, 1994; Lea et al., 1999). For each sex, we estimate:

$$\ln\left[\frac{P\left(Y_{i}=k\right)}{P\left(Y_{i}=0\right)}\right]=\alpha_{k}+\beta_{k}*v_{1i}+\gamma_{k}*h_{i1}+\delta_{k}{Χ}_{i1},k\in \left\{-1,+1\right\}\text{,}$$

where $v_{1i}$ is baseline work-hour volatility, $h_{i1}$ indicates baseline SRH (five-category measure); and ${Χ}_{i1}$ is the Year-1 covariates vector (more information is provided in Appendices A & B).

**Expanded model (less truncated sample; *N* = 61,131).** To reduce employment-based truncation, the expanded specification reintroduces 6069 respondents whose Year-2 status reflects HRWL—limiting, suspending, or withdrawing from standard employment for their own health reasons—and treats HRWL as an additional adverse transition state rather than attrition. The multinomial outcome therefore has four categories—HRWL (−2), deteriorated (−1), stable (0), improved (+1)—and we re-estimate the same weighted multinomial logit separately by sex with $Y_{i}$ ∈{−2, −1, +1}. This design does not identify counterfactual causal effects, but it makes health-driven exit from work observable and broadens inference beyond continuously employed “survivors.”

## 4 Results

### 4.1 Baseline model results

The estimates from our models are pooled across pre-pandemic, pandemic, and post-pandemic periods and therefore reflect average associations across potentially heterogeneous labor-market contexts. Table 2 summarizes the multinomial logit estimates on continuously employed workers only with the baseline modeling. It predicts the three-category health transition outcome separately for men (Models 1–3) and women (Models 4–6). We present the models sequentially: Models 1 and 4 include work-hour volatility and basic demographic controls; Models 2 and 5 add weekly work hours and other socio-economic control variables; and Models 3 and 6, our preferred specifications, add the interaction between volatility and weekly work hours.

**Table 2:** Baseline transition models: Analyzing 3-category health transition (weighted, partial table).

	Multinomial Logit Coefficients for Health Transition between ASEC Year 1 and Year 2					
	Males			Females		
	(1)	(2)	(3)	(4)	(5)	(6)
***Health Deterioration (***$Y_{i}=-1$***) vs. Stable Health (***$Y_{i}=0$)						
Year-1 Work-Hour Volatility	.27∗	.225	−.772∗	.001	−.042	−.172
	(.124)	(.125)	(.336)	(.131)	(.132)	(.324)
Year-1 Health Status	.964∗∗∗	1.022∗∗∗	1.022∗∗∗	.992∗∗∗	1.046∗∗∗	1.046∗∗∗
	(.027)	(.027)	(.027)	(.028)	(.029)	(.029)
Weekly Work Hours		−.001	−.007∗		.000	−.001
		(.002)	(.003)		(.003)	(.003)
Year 1 Volatility × Weekly Work Hours			.027∗∗			.004
			(.009)			(.009)
Constant	−5.214∗∗∗	−5.236∗∗∗	−4.985∗∗∗	−5.216∗∗∗	−5.11∗∗∗	−5.077∗∗∗
	(.151)	(.198)	(.212)	(.161)	(.218)	(.231)
***Health Improvement (***$Y_{i}=+1$***) vs. Stable Health (***$Y_{i}=0$***)***						
Year-1 Work-Hour Volatility	−.08	−.061	−.359	.128	.153	−.325
	(.144)	(.145)	(.407)	(.142)	(.146)	(.408)
Year-1 Health Status	−1.368∗∗∗	−1.399∗∗∗	−1.399∗∗∗	−1.377∗∗∗	−1.396∗∗∗	−1.396∗∗∗
	(.025)	(.026)	(.026)	(.026)	(.027)	(.027)
Weekly Work Hours		−.001	−.003		.002	−.002
		(.003)	(.003)		(.003)	(.004)
Year 1 Volatility × Weekly Work Hours			.008			.014
			(.01)			(.012)
Constant	5.087∗∗∗	4.911∗∗∗	4.986∗∗∗	5.093∗∗∗	4.842∗∗∗	4.961∗∗∗
	(.143)	(.203)	(.221)	(.149)	(.226)	(.242)
Observations	28,968	28,968	28,968	26,094	26,094	26,094
Pseudo *R*^2^	.163	.169	.169	.167	.172	.172

∗∗∗*p* < 0.001, ∗∗*p* < 0.01, ∗*p* < 0.05.

Notes.

1. Entries are multinomial logit coefficients (log-relative-risk coefficients), with stable health as the reference outcome; relative risk ratios ("RRRs" in Stata's terminology) are not reported in the table but are discussed in the main text for ease of interpretation.

2. Standard errors are shown in parentheses. All reported statistical tests are two-tailed.

3. All models adjust for baseline five-category SRH, age, marital status, race/ethnicity, number of children and age of the youngest child in the household, educational attainment, industry, occupation, and period indicators. Models 2 and 5 additionally include weekly work hours as a key control variable. Models 3 and 6 further add the interaction between work-hour volatility and weekly work hours. Full table is available in Appendix B.

Among men, the nested specifications indicate that the volatility–health association should be interpreted in relation to the level of weekly work hours. In Model 1, work-hour volatility is positively associated with health deterioration (*β* = .270, *p* = 0.030). Model 2 adds weekly work hours as a key control variable, providing an intermediate specification before allowing the volatility association to vary by work-hour level (*β* = .225). In the fully adjusted model (Model 3), both the volatility term (*β* = −.772, p = 0.021) and the interaction between volatility and work hours (*β* = .027, *p* = 0.002) are statistically significant at the .05 level. This pattern indicates that the association between volatility and health deterioration varies systematically with the level of work hours: at lower levels of work hours, volatility is less strongly associated with deterioration (and may even be weakly negative), whereas at higher levels of work hours, the association becomes increasingly positive and amplified. By contrast, volatility shows no significant association with health improvement *(*$Y_{i}=+1)$ among men in any specification, indicating that its effect in the baseline models is specific to heightened risk of deterioration.

Among women, however, the corresponding coefficients are small (Models 4–6: *β* = .001, −.042, −.172; RRR = 1.000, .959, .842) and statistically indistinguishable from zero (*p* > 0.05) in the health deterioration model, regardless of covariate specification. Similarly, in the health improvement model, the volatility indicator also yields small coefficients (Models 4–6: *β* = .128, .153, −.325; RRR = 1.136, 1.165, 0.722) without statistical significance (*p* > 0.05). Table 2 reports selected coefficients only, and the full set of covariate estimates is provided in “Appendix B: Full Tables”, Table B2.

Because the fully adjusted models (Table 2, Models 3 and 6) include an interaction between work-hour volatility and weekly work hours, the coefficient on volatility alone does not represent a uniform effect across all workers and may therefore be misleading if interpreted in isolation. To better characterize the substantive pattern, we turn to post-estimation predicted probabilities.

**Fig. 1:**
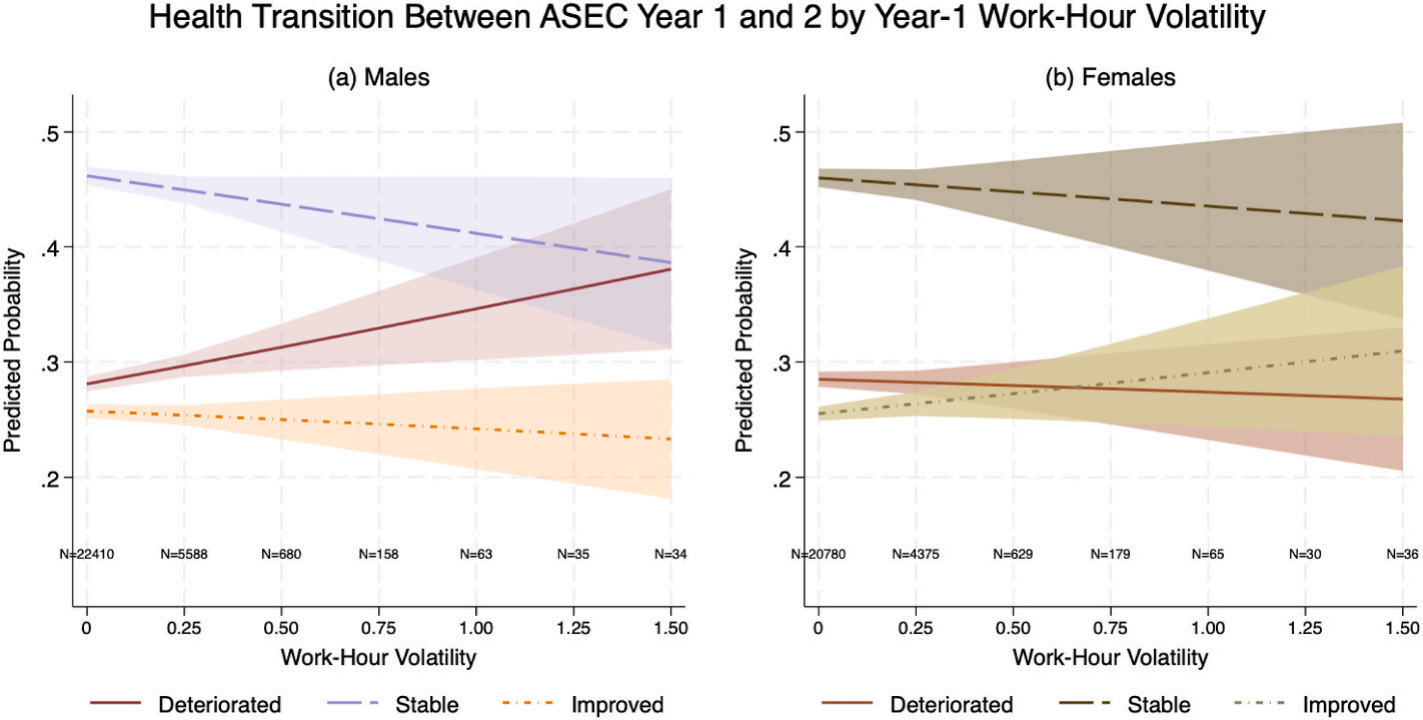
Predicted probability plot based on baseline transition models (3-category health-transition outcomes). Source: IPUMS CPS ASEC 2016–2024, March observations only, analytical samples of employed workers, *N* = 55,062 individuals. Notes: The figure plots model-predicted probabilities (not coefficients or relative risk ratios) of each health-transition outcome—deteriorated, stable, and improved health—from the fully adjusted multinomial logit models in Table 2 (Models 3 and 6), separately for men and women, across values of Year-1 work-hour volatility. The predicted values are computed from the full model specification, including the interaction between volatility and weekly work hours. Sample sizes shown above the x-axis use non-overlapping volatility zones centered on each labeled value except for zero, the lower bound. For example, *v* = 1.00 counts observations with 0.875 ≤ *v* < 1.125, and *v* = 1.50 counts observations with *v* ≥ 1.375. At each value of volatility, the three predicted probabilities within each panel sum to 1. Shaded bands represent 95% confidence intervals. All other covariates (including weekly work hours) are held at their mean values, and all other model settings are the same as in Table 2, Models 3 and 6.

Fig. 1 presents post-estimation predicted probabilities from the fully adjusted multinomial logit models, along with their 95% confidence intervals. The x-axis shows values of Year-1 work-hour volatility, while the y-axis shows the predicted probability of each possible Year-1 to Year-2 health transition outcome. The three outcome categories are: deteriorated health (Year-2 health is worse than Year 1), stable health (no change in health between the two interviews), and improved health (Year-2 health is better than Year 1). Because these three outcomes are mutually exclusive in the baseline model, their predicted probabilities at any given volatility value always sum to 1 within each sex-specific panel.

Among men (Panel A), the predicted probability of health deterioration rises steadily with volatility, increasing from about .28 (28%) at the lowest volatility (*v* = 0, meaning no hour change at all across months) to about .38 (38%) at *v* = 1.5, a level indicating extremely large fluctuations in adjacent-month hours. Over the same range, the predicted probability of stable health declines steadily, from about .46 (46%) to .38 (38%), although the confidence intervals widen and overlap with those of deterioration at higher levels of volatility. Further, the predicted probability of improved health remains the flattest of the three categories, ranging only from about .23 (23%) to .26 (26%). This pattern suggests that higher volatility mainly shifts men's probabilities from stable health toward health deterioration, rather than altering the likelihood of health improvement.

No clear pattern is observed among women (Panel B). The predicted probabilities of health deterioration and improvement change only slightly as volatility increases, and the confidence intervals are wide and heavily overlapping. Although there are minor directional shifts, the uncertainty around the estimates makes it difficult to distinguish these patterns or to draw firm conclusions about the association. Although the predicted probability of stable health is somewhat separated from the other two outcomes, its confidence intervals also become extremely wide at higher levels of volatility, accompanied by the minor probability changes from .46 (46%) to .42 (42%) over the entire volatility range of 1.5, making it difficult to identify a clear linear relationship. These patterns are consistent with the insignificant volatility coefficients in Models 4 and 6 of Table 2 and therefore do not support a substantive conclusion about the association between volatility and baseline SRH transition among continuously employed women.

Overall, the evidence suggests that greater instability in working hours is associated with subsequent health deterioration for men, especially at higher weekly work hours, but not for women. Among women, volatility is not significantly associated with either SRH deterioration or improvement within baseline employed-only models. This pattern raises a natural question: if work-hour volatility does not emerge as health deterioration among women within employed-only models, where—if anywhere—might its consequences become observable outcomes? The next subsection addresses this question by examining an expanded outcome that brings health-related labor-force limitation into view.

### 4.2 Expanded model results

Prior literature suggests that men and women respond differently to work-related health strain: after an occupational injury or illness, women are often more likely than men to interrupt, scale back, or suspend paid work, and the duration and consequences of that interruption differ by gender (Boden & Galizzi, 2003; Macpherson et al., 2019; Timp et al., 2024). As a result, the health consequences of unstable work time may not always appear the same for men and women. These consequences may be observed differently within this study's design: for men, as a gradual decline in self-rated health while still employed, and for women, as outcomes that become more visible through work limitation, such as restriction of or withdrawal from standard, active employment for health or medical reasons.

Guided by this possibility, we now turn from the baseline models in Section 4.1 to an expanded specification that (a) restores workers who, by Year 2, report limiting, suspending, or leaving work for their own health or medical reasons, and (b) treats that health-related work limitation (HRWL) as an additional transition outcome. This expanded framework asks whether volatility predicts not only self-rated health deterioration among those who remain employed, but also the emergence of health-related restriction of, suspension from, or withdrawal from standard active employment—changes that become observable only in Year 2.

Model results in Table 3 show that adding the health-related work limitations (HRWL) category ($Y_{i}=-2$**)** changes the pattern for women. When HRWL is included as an outcome, work-hour volatility is significantly associated with women's transition into health-related work limitation, an outcome that reflects health-related exit or restriction within the observed follow-up period (Model 2: *β* = −1.216, RRR = .296, *p* = 0.041). In addition, the interaction between volatility and weekly work hours is positive and statistically significant (*β* = .054, RRR = 1.056. *p* = 0.001), indicating that the association between volatility and HRWL varies with the level of work hours within this observation period. Specifically, at lower levels of work hours, the association is weaker (and may be negative), whereas at higher levels of work hours it becomes more positive, suggesting that the risk of entering HRWL under volatile schedules is concentrated among women working longer hours. By contrast, volatility remains unrelated to moderate SRH deterioration or improvement among women. This pattern is consistent with the expectation that, for women, the consequences of work-time instability may be more readily observed in this study through health-related work limitation —a transition that also reflects how health problems translate into labor-force changes—rather than through changes in self-rated health alone within the observed follow-up period.

**Table 3:** Expanded transition model: Analyzing 4-category health transition (weighted, partial table).

	(1)	(2)
	Males	Females
***Health-Related Work Limitation***$\left(Y_{i}=-2\right)$***vs. Stable Health (***$Y_{i}=0$***)***		
Year-1 Work-Hour Volatility	−1.108	−1.216∗
	(.73)	(.596)
Year 1 Volatility × Weekly Work Hours	.04∗	.054∗∗
	(.019)	(.017)
Year-1 Health Status	−.502∗∗∗	−.512∗∗∗
	(.083)	(.066)
Weekly Work Hours	−.041∗∗∗	−.036∗∗∗
	(.01)	(.006)
Constant	−1.052	−.653
	(.619)	(.488)

***Health Deterioration (***$Y_{i}=-1$***) vs. Stable Health (***$Y_{i}=0$***)***		
Year-1 Work-Hour Volatility	−.744∗	.11
	(.319)	(.252)
Year 1 Volatility × Weekly Work Hours	.027∗∗	−.001
	(.008)	(.007)
Year-1 Health Status	1.011∗∗∗	1.02∗∗∗
	(.026)	(.027)
Weekly Work Hours	−.009∗∗	.003
	(.003)	(.003)
Constant	−4.887∗∗∗	−5.049∗∗∗
	(.202)	(.211)

***Health Improvement (***$Y_{i}=+1$***) vs. Stable Health (***$Y_{i}=0$***)***		
Year-1 Work-Hour Volatility	−.046	−.081
	(.358)	(.311)
Year 1 Volatility × Weekly Work Hours	.000	.008
	(.009)	(.01)
Year-1 Health Status	−1.378∗∗∗	−1.396∗∗∗
	(.025)	(.025)
Weekly Work Hours	.000	.000
	(.003)	(.003)
Constant	4.781∗∗∗	4.911∗∗∗
	(.206)	(.216)

Observations	31,238	29,893
Pseudo *R*^2^	.158	.159

∗∗∗*p* < 0.001, ∗∗*p* < 0.01, ∗*p* < 0.05.

Notes.

1. Entries are multinomial logit coefficients (log-relative-risk coefficients), with stable health as the reference outcome; relative risk ratios ("RRRs" in Stata's terminology) are not reported in the table but are discussed in the main text for ease of interpretation.

2. Standard errors are shown in parentheses. All reported statistical tests are two-tailed.

3. All models additionally adjust for baseline five-category SRH, age, marital status, race/ethnicity (African American, Hispanic, Asian, Native American, multiracial; reference = non-Hispanic White), number of children and age of the youngest child in the household, educational attainment, work hours and their interaction with volatility, industry, occupation, and period indicators (pre-, mid-, and post-COVID).

For men, however, adding HRWL $\left(Y_{i}=-2\right)$ does not substantively alter the pattern observed in the baseline models. Work-hour volatility shows no statistically significant direct association with HRWL (Model 1: *β* = −1.108, RRR = .330, *p* = 0.129), although its interaction with weekly work hours is modestly significant (*β* = .04, RRR = 1.041, *p* = 0.035), indicating some variations by work-hour level. At the same time, volatility continues to predict self-rated health deterioration, with both the main volatility term (*β* = −.744, RRR = .475, *p* = 0.020) and its interaction with work hours (*β* = .027, RRR = 1.028, *p* = 0.001) remaining statistically significant. These estimates are highly similar in magnitude to those reported in Model 3 of Table 2, suggesting that the association between volatility and health deterioration among men is robust to the inclusion of health-related work limitation as an additional outcome. Consistent with Table 2, volatility also shows no association with men's likelihood of health improvement across years (Table 3 Model 1: *β* = −.046, RRR = .944, *p* = 0.684). Those results strengthen the interpretation that, among men, the health costs of work-hour volatility are more likely to appear as worsening self-rated health while remaining employed rather than as health-related restriction of employment.

Because the expanded models include volatility × work-hour interactions, we again use post-estimation predicted probabilities to clarify how the likelihood of HRWL, deterioration, stability, and improvement changes as volatility rises.

**Fig. 2:**
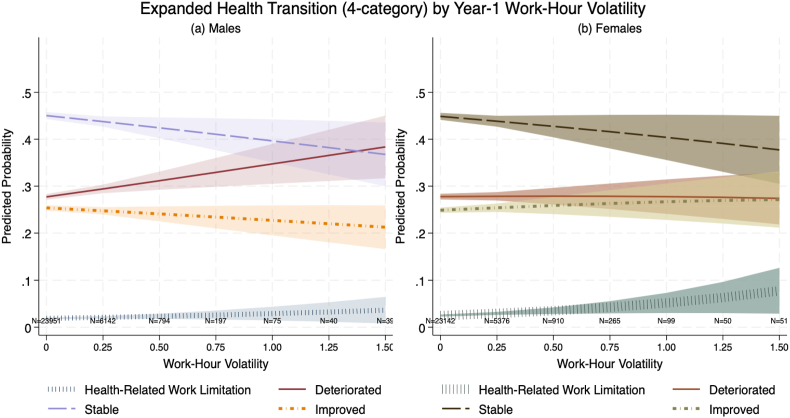
Predicted probability plot based on expanded transition model (4-category health outcomes). Source: IPUMS CPS ASEC 2016–2024, March observations only, *N* = 61,131 individuals ← 55,062 analytical samples of employed workers + 6069 expanded samples of inactive labor due to health limitations.Notes: The figure plots model-predicted probabilities (not coefficients or relative risk ratios) of each expanded health-transition outcome—health-related work limitation, deteriorated health, stable health, and improved health—from the fully adjusted multinomial logit models in Table 3 , separately for men and women, across values of Year-1 work-hour volatility. The predicted values are computed from the full model specification, including the interaction between volatility and weekly work hours. Sample sizes shown above the x-axis use non-overlapping volatility zones centered on each labeled value except for zero, the lower bound. For example, *v* = 1.00 counts observations with 0.875 ≤ *v* < 1.125, and *v* = 1.50 counts observations with *v* ≥ 1.375. At each value of volatility, the four predicted probabilities within each panel sum to 1. Shaded bands represent 95% confidence intervals. All other covariates (including weekly work hours) are held at their mean values, and all other model settings are the same as in Table 3.

Fig. 2 shows a similar pattern and confirms the expanded-model results reported in Table 3. Among men (Panel A), the predicted probability of health deterioration increases steadily as volatility rises, from about .277 (27.7%) at *v* = 0 to about .384 (38.4%) at *v* = 1.5, while the predicted probability of stable health declines from roughly .450 (45.0%) to .368 (36.8%). The predicted probability of improved health decreases only modestly (from about 25.4% to 21.3%), and the probability of health-related work limitation remains very low throughout, rising only from about .019 (1.9%) to .036 (3.6%). Thus, for men, the expanded model confirms that higher volatility is associated primarily with a redistribution from stable health toward health deterioration, rather than with health-related withdrawal from employment.

Among women (Panel B), the expanded-model probabilities suggest a clearer directional reallocation of stable health toward HRWL, a pattern not observable in the baseline models. As work-hour volatility rises, the predicted probability of stable health declines from about .449 (44.9%) at *v* =0, to about .377 (37.7%) at v=1.5, although the confidence intervals are wide. Across the same range, the predicted probability of health deterioration remains largely flat (from about .278 to .274), while the probability of health improvement increases only modestly (from about .249 to .271), with substantial overlap in their confidence intervals and no clear separation.

The most notable adverse movement is in HRWL, whose predicted probability increases from about 2.5% to 7.7%, as volatility rises from 0 to 1.5. Although the confidence intervals widen substantially at higher levels of volatility, this upward pattern stands out relative to the relatively flat trajectories of deterioration and improvement. The post-estimation probability predictions are broadly consistent with the coefficient results: among women, greater volatility is less clearly expressed as worsening self-rated health alone than as movement away from stable employment and into health-related work limitation. While a 7.7% probability may appear modest in absolute terms, it is comparable to one-year health-attributed employment disruptions observed after major health shocks such as myocardial infarction or breast cancer (Hassett et al., 2009; Warraich et al., 2018), underscoring its potential real-world significance.

To conclude, our baseline and expanded models suggest that work-hour volatility is associated with distinct observable health patterns for men and women. For men, volatility is primarily associated with health deterioration while remaining employed. For women, the association becomes visible primarily through HRWL, rather than through SRH deterioration among continuously employed workers. Incorporating HRWL thus appears to reflect a dimension that employed-only analyses would otherwise miss.

## 5 Discussion & conclusion

### 5.1 Discussion

This study adds new evidence to the emerging view that precarious work—including its temporal dimension—is itself a social correlate of health (Benach et al., 2014; Frank et al., 2023). Rather than focusing on contract types or employment insecurity, it captures temporal precarity directly as month-to-month volatility in usual weekly hours for the same worker.

Two core findings stand out. Among continuously employed workers, greater volatility is associated with subsequent health deterioration for men. When the outcome is expanded to include health-related work limitation—leaving, suspending, or reducing work for one's own health reasons— volatility instead emerges as a strong predictor of withdrawal from regular employment for women. Thus, instability in work hours is not merely a scheduling inconvenience but a population-level—and gendered—health risk within the observed CPS sample and roughly one-year follow-up window. This pattern is consistent with prior evidence that gender structures exposure to adverse work conditions and the terms on which workers remain employed as health deteriorates (Campos-Serna et al., 2013; Read & Gorman, 2010).

One possible interpretation of the gender difference is that the same work-condition exposure may become visible through different health outcomes, because women and men differ in morbidity, functional limitation, and the health conditions they disproportionately experience. Prior research shows that women tend to carry a heavier burden of nonfatal but disabling chronic conditions and functional limitations, whereas men experience more severe or mortality-linked conditions (Case & Paxson, 2005; Crimmins et al., 2019; Elgaddal Kramarow Weeks & Reuben, 2024). This may help explain why, in our data, work-hour instability is more readily expressed as worsening self-rated health among men, while among women it may become visible through health-related work limitation. This interpretation should remain cautious, however, because self-rated health may also reflect different underlying constellations of symptoms and functional constraints (Case & Paxson, 2005; Zajacova et al., 2017).

More importantly, our results speak directly to a longstanding methodological challenge in occupational and social epidemiology: the healthy worker survivor effect. Conventional approaches often assess health only among those who remain employed, implicitly censoring workers who reduce hours, miss work, or leave employment for health reasons (Arrighi & Hertz-Picciotto, 1994; Buckley et al., 2015; Lea et al., 1999). Replicating this logic, the employed-only models in this study reveal a clear volatility–health deterioration association only among men. Once health-related work limitation is treated as part of the observed outcome process—rather than as attrition, a different pattern becomes visible for women. Although the evidence is observational, it aligns with prior research showing that women are more likely than men to interrupt or suspend paid work in response to health strain or injury (Boden & Galizzi, 2003; Macpherson et al., 2019; Timp et al., 2024). More broadly, these findings suggest that employed-only analyses may understate part of the observability of work-hour instability, when health-related reductions in or withdrawal from work are excluded from the outcome process.

These findings invite a more cautious reconsideration of how occupational health research conceptualizes exposure, harm, and adaptation in precarious labor regimes. They reinforce arguments that temporal precarity should be treated as structural health exposure, not an individual lifestyle choice (Cai, 2024a, 2024b; Cai & Peck, 2022; Schneider & Harknett, 2019). Although the CPS does not identify who initiated schedule changes, prior research links unstable hours to managerial control over workers’ time, earnings instability, sleep disruption, caregiving strains, and limited bargaining power (Gerstel & Clawson, 2018; Golden and Kim, 2017; Lambert et al., 2019; Lu & Leicht, 2025), In the present study, such instability is associated with worsening self-rated health among men and health-related work limitation among women, suggesting possible downstream implications for earnings, benefits, health insurance, and health disparities.

Although this study does not test mechanisms directly, prior research suggests several plausible pathways through which unstable work hours may affect health. Unstable schedules are linked to psychological distress, sleep disruption, and chronic fatigue (Harknett et al., 2020); large swings in hours are associated with volatile earnings and material hardship (Thomas et al., 2022) within the observed transition; and irregular or reduced hours can erode access to employer-linked benefits, including health insurance and timely treatment (McWilliams, 2009). Because our measure captures realized fluctuations rather than their sources, the observed associations should be interpreted as reflecting the consequences of work-hour instability broadly, not any single scheduling mechanism. These proposed mechanisms come from prior research, not from the present analysis. Still, the findings are consistent with research arguing that temporal precariousness matters for health and health inequality (Hajat et al., 2024; Schneider & Harknett, 2019).

### 5.2 Limitations

Several limitations warrant note. First, the linked CPS design provides only two annual health observations one year apart; thus, the analysis focuses on one-year transitions and cannot estimate longer-run hazard/duration processes or within-person trajectories that require more repeated health measures over years (Chevrier et al., 2012; Ko et al., 2024). Second, SRH—while predictive of morbidity and mortality—is subjective and socially patterned, with reporting potentially varying by gender and socioeconomic context; it is also sensitive to recent symptoms and acute shocks that CPS does not measure (Ferraro & Kelley-Moore, 2001; Idler & Benyamini, 1997; Jylhä, 2009; Read & Gorman, 2010; Virtanen et al., 2004; Zajacova et al., 2017).

Third, although we model HRWL as an outcome, CPS cannot observe medical severity, disability adjudication, employer accommodations, or whether exits are temporary versus permanent; HRWL therefore indicates meaningful work-limiting deterioration but cannot be ranked by medical or clinical severity. In addition, because HRWL is observed only at a single one-year follow-up, its visibility may be shaped by the timing of gendered exit and recovery processes. Prior research suggests that women may recover more slowly than men in the short term from sickness absence or work disability, even when longer-run gender differences are smaller (Macpherson et al., 2019). As a result, women's health-related work restriction or exit may be more likely to remain observable at the Year-2 interview, whereas some men may experience shorter-duration work limitation and return to standard employment before follow-up, making that health outcome invisible under our current research design. We therefore interpret the current HRWL findings as suggestive and encourage future research using longer panels and richer physical and mental health measures.

Fourth, the exposure captures a core quantitative dimension of temporal precarity—month-to-month volatility in usual weekly hours (Cai, 2024a, 2024b; Cai & Mattingly, 2025; Cai & Peck, 2022)—but lacks qualitative information on schedule control (e.g., who initiated changes, desirability, and counterfactual preferences). This volatility measure should also be recognized as a summary indicator of month-to-month fluctuation in work hours and does not provide information on the process through which that instability is produced.^16^

Finally, the study remains observational: workers are not randomly assigned to volatile hours, and unmeasured factors (e.g., employer sorting, informal performance concerns, or early symptoms not captured by baseline SRH) may confound associations. While expanding the sample and outcome to include HRWL reduces employment-based truncation and makes health-driven withdrawal observable, it addresses only the censoring component of the healthy-worker survivor problem and does not establish causal effects; stronger causal designs or quasi-experimental variation in volatility are important directions for future research. In addition, because the analysis spans pre-pandemic, pandemic, and post-pandemic periods, observed volatility may reflect different underlying processes across these contexts (e.g., demand shocks versus routine scheduling practices), which this study does not separately identify.

### 5.3 Conclusion

This study suggests that temporal precarity—the organization and stability of hours—may be an important dimension of occupational health risk, as observed in short-run transitions within CPS-linked data. Incorporating the temporal organization of work—how many hours are worked, when they occur, and how stable they are—into the occupational health framework may help clarify why similar labor-market exposures are associated with different observed health trajectories for men and women and how scheduling practices may relate to both health inequality and labor-force attachment. This also has policy implications within the study's observation window: it aligns with emerging evidence from fair workweek regulations^17^ requiring advance notice of schedules, predictability pay for last-minute employer-initiated changes, and limits on short-turnaround shifts, all of which have been linked to improved schedule predictability and worker well-being (Ananat et al., 2022; Harknett et al., 2021). It also lends support to federal legislative efforts such as the Schedules That Work Act^18^, which would give employees the right to request more predictable and stable schedules and require compensation for disruptive short-notice changes (DeLauro, 2019; Miller, 2014).

The analysis contributes a design-based strategy for reducing employment-based truncation related to the healthy worker survivor effect: to follow workers even when they reduce or exit standard employment for health reasons and treat health-related work limitations (HRWL) as an adverse health transition, capturing health-related changes in labor-force participation that would otherwise be censored, rather than sample attrition. This expanded modeling specification, as observed in CPS-linked data within short-run transitions, makes visible a dimension of health-related change that employed-only models can miss, even though it does not eliminate selection bias or recover causal effects. Future work can build on this approach by refining measurement of health-related work limitation among those who remain employed but medically restrict their work, extending analyses to longer panels and higher-frequency scheduling data, and pairing selection-aware outcome constructions with established causal designs to better capture cumulative exposure and time-varying processes.

## Ethical statement

This study uses publicly available, de-identified secondary data from the IPUMS CPS-ASEC and CPS Basic Monthly files (Integrated Public Use Microdata Series). The data contain no personally identifiable information and involve no direct interaction with human subjects. Under U.S. federal regulations, analysis of de-identified public-use data does not constitute human subjects research and is therefore exempt from institutional review board (IRB) oversight.

## Declaration of generative AI and AI-assisted technologies in the manuscript preparation process

During the preparation of this work, the authors used Grammarly and ChatGPT to improve the clarity, readability, and language of the manuscript. After using these tools, the authors reviewed and edited the content as needed and take full responsibility for the content of the published article.

## CRediT authorship contribution statement

**Sinyee Qianyi Lu:** Conceptualization, Data curation, Formal analysis, Investigation, Methodology, Visualization, Writing – original draft, Writing – review & editing. **Tim F. Liao:** Conceptualization, Funding acquisition, Methodology, Supervision, Writing – review & editing.

## Declaration of competing interests

The authors declare that they have no known competing financial interests or personal relationships that could have appeared to influence the work reported in this paper.

## Figures and Tables

**Appendix Table 1:** Variable Overview: Continuously Employed Samples Only, Individual Weights Applied

Variable Name	Males (*N* = 28,968)				Females (*N* = 26,094)			
	S.D.	Min.	Mean/Med.	Max.	S.D.	Min.	Mean/Med.	Max.
Work Hours in Year 1								
Weekly Work Hours (on ASEC Month, in Year 1)	7.69	1	42.19	99	7.30	1	39.90	99
Within-Individual Month-to-Month Volatility	.14	0	.07	3	.14	0	.07	3

Self-Rated Health (SRH)								
Five-Category SRH in Year 1: 1poor/2fair/3good/4very good/5excellent	.88	1	4	5	.89	1	4	5
Five-Category SRH in Year 2: 1poor/2fair/3good/4very good/5excellent	.88	1	4	5	.88	1	4	5
Three-Category SRH Transition Between Year 1 & 2: +1 improved/0 stable/-1 deteriorated	.74	−1	0	1	.74	−1	0	1

Control Variables in Year 1								
Age	12.02	18	42.89	64	11.95	18	43.95	64
Married	.49	0	.60	1	.49	0	.58	1
White	.43	0	.75	1	.44	0	.75	1
Black/African American	.25	0	.07	1	.29	0	.09	1
Hispanic	.32	0	.11	1	.29	0	.09	1
Asian American	.22	0	.05	1	.22	0	.05	1
Native American	.09	0	.01	1	.09	0	.01	1
Multi-Racial	.11	0	.01	1	.12	0	.01	1
Number of Children in the Household	1.10	0	.76	9	1.03	0	.74	9
Youngest Child: No Own Child	.46	0	.70	1	.45	0	.72	1
Youngest Child: Aged 0-4	.31	0	.11	1	.28	0	.08	1
Youngest Child: Aged 5-17	.39	0	.19	1	.40	0	.20	1
Education: Less Than High School	.22	0	.05	1	.17	0	.03	1
Education: High School Graduate	.45	0	.28	1	.41	0	.21	1
Education: Some College	.43	0	.25	1	.44	0	.26	1
Education: Bachelor's Degree	.45	0	.27	1	.46	0	.30	1
Education: Graduate Degree	.35	0	.15	1	.40	0	.20	1
Industry: Primary	.15	0	.02	1	.10	0	.01	1
Industry: Secondary	.44	0	.27	1	.29	0	.09	1
Industry: Tertiary -- Material-Related Services	.44	0	.27	1	.38	0	.18	1
Industry: Tertiary -- Information & Human Services	.50	0	.44	1	.45	0	.72	1
Occupation: Service Position	.44	0	.26	1	.49	0	.38	1
Occupation: Production Position	.47	0	.32	1	.24	0	.06	1
Occupation: Management & Professional Position	.49	0	.42	1	.50	0	.56	1

**Appendix Table 2:** Gender-Specific Baseline Transition Model (Weighted): Predicting 3-Category Health Transition between ASEC Year 1 and 2

	Multinomial Logit Coefficients for Health Transition between ASEC Year 1 and Year 2					
	(1)	(2)	(3)	(4)	(5)	(6)
	Males			Females		
***Health Deterioration (***$Y_{i}=-1$***) vs. Stable Health (***$Y_{i}=0$***)***						
Year-1 Work-Hour Volatility	.27∗	.225	−.772∗	.001	−.042	−.172
	(.124)	(.125)	(.336)	(.131)	(.132)	(.324)
Year-1 Health Status	.964∗∗∗	1.022∗∗∗	1.022∗∗∗	.992∗∗∗	1.046∗∗∗	1.046∗∗∗
	(.027)	(.027)	(.027)	(.028)	(.029)	(.029)
Mid-Covid: 2020 to 2022	−.012	.012	.013	−.025	.007	.007
	(.042)	(.042)	(.042)	(.044)	(.044)	(.044)
Post-Covid: 2022 to 2024	.048	.072	.071	.057	.09∗	.09∗
	(.042)	(.042)	(.042)	(.044)	(.044)	(.044)
Age	.015∗∗∗	.016∗∗∗	.016∗∗∗	.011∗∗∗	.01∗∗∗	.01∗∗∗
	(.002)	(.002)	(.002)	(.002)	(.002)	(.002)
Married	−.189∗∗∗	−.132∗∗	−.13∗∗	−.076	−.039	−.039
	(.044)	(.045)	(.045)	(.041)	(.041)	(.041)
African American	.289∗∗∗	.232∗∗∗	.228∗∗	.284∗∗∗	.25∗∗∗	.25∗∗∗
	(.069)	(.07)	(.07)	(.062)	(.062)	(.062)
Hispanic	.294∗∗∗	.138∗∗	.136∗	.247∗∗∗	.12∗	.12∗
	(.05)	(.054)	(.054)	(.056)	(.059)	(.059)
Asian	.085	.195∗∗	.193∗∗	.155∗	.228∗∗	.228∗∗
	(.07)	(.071)	(.071)	(.074)	(.076)	(.076)
Native	.136	.081	.074	.387∗	.274	.275
	(.176)	(.174)	(.174)	(.177)	(.176)	(.176)
Multi-Racial	.21	.168	.161	.193	.148	.148
	(.148)	(.15)	(.151)	(.152)	(.154)	(.154)
Number of Children in the Household	.039+	.022	.022	.085∗∗	.068∗∗	.068∗∗
	(.023)	(.023)	(.023)	(.025)	(.026)	(.026)
Youngest Child: Aged 0-4	−.112	−.062	−.058	−.182∗	−.103	−.103
	(.07)	(.071)	(.071)	(.076)	(.077)	(.077)
Youngest Child: Aged 5-17	−.025	.026	.028	−.105	−.052	−.053
	(.059)	(.059)	(.059)	(.06)	(.06)	(.06)
Education: High School Graduate		−.093	−.092		−.072	−.072
		(.082)	(.082)		(.104)	(.104)
Education: Some College		−.308∗∗∗	−.308∗∗∗		−.24∗	−.239∗
		(.084)	(.085)		(.104)	(.104)
Education: Bachelor's Degree		−.49∗∗∗	−.488∗∗∗		−.461∗∗∗	−.46∗∗∗
		(.089)	(.089)		(.108)	(.108)
Education: Graduate Degree		−.589∗∗∗	−.586∗∗∗		−.711∗∗∗	−.71∗∗∗
		(.098)	(.099)		(.113)	(.113)
Weekly Work Hours (ASEC One-Month Measure)		−.001	−.007∗		.000	−.001
		(.002)	(.003)		(.003)	(.003)
Work Hours × Volatility			.027∗∗			.004
			(.009)			(.009)
Industry: Primary		.172	.175		.06	.06
		(.108)	(.109)		(.176)	(.176)
Industry: Secondary		−.004	−.004		−.093	−.092
		(.049)	(.049)		(.07)	(.071)
Industry: Tertiary -- Material-Related Services		.122∗∗	.124∗∗		−.026	−.026
		(.047)	(.047)		(.051)	(.051)
Occupation: Service Position		.007	.005		.023	.023
		(.051)	(.051)		(.045)	(.045)
Occupation: Production Position		.108∗	.106∗		.165	.164
		(.054)	(.054)		(.09)	(.09)
Constant	−5.214∗∗∗	−5.236∗∗∗	−4.985∗∗∗	−5.216∗∗∗	−5.11∗∗∗	−5.077∗∗∗
	(.151)	(.198)	(.212)	(.161)	(.218)	(.231)
***Health Improvement (***$Y_{i}=+1$***) vs. Stable Health (***$Y_{i}=0$***)***						
Year-1 Work-Hour Volatility	−.08	−.061	−.359	.128	.153	−.325
	(.144)	(.145)	(.407)	(.142)	(.146)	(.408)
Year-1 Health Status	−1.368∗∗∗	−1.399∗∗∗	−1.399∗∗∗	−1.377∗∗∗	−1.396∗∗∗	−1.396∗∗∗
	(.025)	(.026)	(.026)	(.026)	(.027)	(.027)
Mid-Covid: 2020 to 2022	−.022	−.038	−.038	−.059	−.066	−.065
	(.047)	(.047)	(.047)	(.048)	(.049)	(.049)
Post-Covid: 2022 to 2024	−.145∗∗	−.164∗∗∗	−.164∗∗∗	−.143∗∗	−.153∗∗	−.152∗∗
	(.047)	(.048)	(.048)	(.05)	(.05)	(.05)
Age	−.016∗∗∗	−.016∗∗∗	−.016∗∗∗	−.018∗∗∗	−.017∗∗∗	−.017∗∗∗
	(.002)	(.002)	(.002)	(.002)	(.002)	(.002)
Married	.065	.047	.047	.205∗∗∗	.201∗∗∗	.201∗∗∗
	(.05)	(.05)	(.05)	(.045)	(.045)	(.045)
Black	.06	.089	.087	−.143∗	−.128	−.128
	(.082)	(.082)	(.082)	(.07)	(.07)	(.07)
Hispanic	.018	.111	.11	.015	.074	.073
	(.056)	(.059)	(.059)	(.062)	(.064)	(.064)
Asian	−.114	−.161∗	−.161∗	−.036	−.05	−.049
	(.079)	(.081)	(.081)	(.084)	(.085)	(.085)
Native	−.382∗	−.347	−.35	−.071	−.044	−.04
	(.182)	(.182)	(.182)	(.177)	(.178)	(.178)
Multi-Racial	−.079	−.053	−.055	.009	.02	.019
	(.171)	(.172)	(.172)	(.172)	(.171)	(.171)
Number of Children in the Household	−.014	−.006	−.007	.035	.041	.041
	(.026)	(.026)	(.026)	(.028)	(.028)	(.028)
Youngest Child: Aged 0-4	.098	.085	.085	−.034	−.038	−.039
	(.078)	(.078)	(.078)	(.086)	(.086)	(.086)
Youngest Child: Aged 5-17	.046	.028	.029	−.03	−.035	−.036
	(.066)	(.066)	(.066)	(.066)	(.067)	(.067)
Education: High School Graduate		.245∗∗	.245∗∗		.112	.113
		(.094)	(.094)		(.123)	(.123)
Education: Some College		.317∗∗∗	.317∗∗∗		.168	.168
		(.096)	(.096)		(.123)	(.123)
Education: Bachelor's Degree		.441∗∗∗	.441∗∗∗		.307∗	.309∗
		(.101)	(.101)		(.127)	(.127)
Education: Graduate Degree		.441∗∗∗	.442∗∗∗		.2	.203
		(.111)	(.111)		(.133)	(.133)
Weekly Work Hours (ASEC One-Month Measure)		−.001	−.003		.002	−.002
		(.003)	(.003)		(.003)	(.004)
Work Hours × Volatility			.008			.014
			(.01)			(.012)
Industry: Primary		−.012	−.011		.005	.005
		(.128)	(.128)		(.197)	(.197)
Industry: Secondary		.059	.059		.03	.034
		(.054)	(.054)		(.079)	(.079)
Industry: Tertiary -- Material-Related Services		.035	.035		.075	.076
		(.053)	(.053)		(.057)	(.057)
Occupation: Service Position		.000	.000		.021	.02
		(.057)	(.057)		(.05)	(.05)
Occupation: Production Position		−.059	−.059		−.235∗	−.236∗
		(.06)	(.06)		(.103)	(.103)
Constant	5.087∗∗∗	4.911∗∗∗	4.986∗∗∗	5.093∗∗∗	4.842∗∗∗	4.961∗∗∗
	(.143)	(.203)	(.221)	(.149)	(.226)	(.242)

Observations	28,968	28,968	28,968	26,094	26,094	26,094
Pseudo *R*^2^	.163	.169	.169	.167	.172	.172

Standard errors are in parentheses.

∗∗∗*p* < 0.001, ∗∗*p* < 0.01, ∗*p* < 0.05.

## Data Availability

The IPUMS CPS data used in this study are available to registered users through the University of Minnesota's IPUMS CPS platform (cps.ipums.org/cps/). Under the IPUMS CPS user license, the authors cannot redistribute the underlying data or analytical dataset. However, additional details and supporting scripts of the data-cleaning and analysis procedures are available from the authors upon request.

## Appendix A Data and Method Details

### 3.1 Data

We use the Current Population Survey (CPS) Annual Social and Economic Supplement (ASEC) linked to the CPS Basic Monthly files, obtained from the Integrated Public Use Microdata Series (IPUMS), provided by the Institute for Social Research and Data Innovation at the University of Minnesota. IPUMS curates, harmonizes, and disseminates government-produced data collections—such as the CPS^19^, originally collected by the U.S. Census Bureau and the Bureau of Labor Statistics—facilitating within-household linkage across individuals, longitudinal tracking of the same individuals over time, and linkage across different surveys for the same households.

The CPS ASEC is the most comprehensive supplement administered within the CPS across the calendar year. It includes the self-rated health item used in this study to construct between-year health transitions, as well as rich information on income, poverty, social welfare program participation, and health insurance coverage, although the latter measures are not directly analyzed here. These features make the ASEC especially valuable for linking annual health information to the higher-frequency labor-force information contained in the CPS Basic Monthly files. We selected data from 2016 to 2024, while excluding 2018-2019^20^ and 2019-2020,^21^ which generated six annual cohorts (2016–2017 through 2023–2024).

The CPS follows a 4–8–4 sampling rotation: respondents are interviewed for four consecutive months in the baseline year (e.g., January–April), out of sample for the eight subsequent months, and are then reinterviewed in the same four calendar months in the following year before permanently exiting the sample. Using IPUMS longitudinal link keys, we link individuals observed in ASEC at time t (“Year 1”) to the same individuals observed in ASEC in the same calendar month at time *t*+1 (“Year 2”), enabling us to observe within-person health trajectories across two consecutive calendar years, with two four-month observation periods separated by an eight-month interval without observations^22^. While the health outcome is observed only at two time points (ASEC Year 1 and Year 2), work hours are observed repeatedly in the intervening CPS Basic Monthly interviews, providing six to eight within-person observations over the same interval. To construct the key exposure, we attach intervening Basic Monthly interviews between ASEC(*t*) and ASEC (*t*+1), yielding up to eight monthly observations (four from each in-sample spell) to measure within-person month-to-month volatility in weekly work hours. The unit of analysis is therefore the linked individual observed across two consecutive ASEC interviews, with monthly work-hour information used to construct the volatility measure between those interviews. This yields 428,389 linked person-years.

The CPS-ASEC is uniquely suited to this study because it combines (1) nationally representative coverage of the civilian noninstitutional U.S. population, (2) a validated annual self-rated health measure, and (3) monthly labor-force information that allows construction of within-person month-to-month volatility in weekly work hours.^23^ Most longitudinal surveys with richer clinical or biomarker-based health measures (e.g., PSID^24^, Add Health^25^, NLSY^26^, and HRS^27^ lack monthly labor-force data needed to measure short-run work-hour instability, whereas work-focused surveys and modules (e.g., GSS -- Quality of Worklife Module^28^ are typically cross-sectional, so they do not provide repeated within-person self-rated health measures needed to analyze health change. The CPS 4–8–4 rotating panel design therefore uniquely enables linkage of annual health transitions to intervening monthly work-hour volatility in a population-representative framework^29^.

To contract our unique analytical sample, we first retain respondents with validated ASEC interviews in both Year 1 and Year 2 and who appear in both four-month in-sample spells of the CPS 4–8–4 rotation, ensuring complete linkage across the one-year interval. Second, to construct a reliable volatility measure, we require at least three Basic Monthly interviews in each in-sample spell (minimum six total months) with valid weekly hours reported in all applicable months^30^. Individuals with zero hours, “not at work,” or missing weekly hours during intervening months are excluded to avoid conflating employment interruptions with hour-to-hour instability. Third, we restrict to working-age civilians (ages 18–64) who are employed and in the labor force during the observation window, excluding full-time students, active-duty military, unpaid family workers, and individuals reporting part-time work for health-related reasons during the ASEC reference week. Finally, we remove observations with missing values on the outcome, key predictors, or covariates.

Applying these criteria yields an analytic sample of 55,062 individuals from 40,687 households. This baseline sample consists of the foundation of our baseline transition models in Section 4.1 and is consistent with conventional employed-only analytic designs in occupational epidemiology, in which workers are effectively truncated once they reduce hours, change employer, exit employment, or otherwise leave the at-risk group. In later analyses (Section 4.2), we reintroduce an additional 6069 individuals who, by Year 2, report limiting, suspending, or withdrawing from standard employment specifically for their own health or medical reasons. Including these health-limited cases expands the panel to 61,131 individuals and allows me to model those health-related labor-force limitations themselves as adverse health transitions, rather than dropping them when they exit regular employment for reported health reasons.

### 3.2 Measures

#### 3.2.1 Baseline Dependent Variable: Between-Year Health Transition

CPS-ASEC includes a five-category self-rated health (SRH) item—excellent, very good, good, fair, poor^31^—that captures respondents' overall health at the time of interview. SRH is a widely used global health summary measure that reflects respondents’ integrated assessment of physical symptoms and diagnosed conditions, functional limitations, and mental well-being, drawing on information that may not be fully captured by single-domain indicators (Benyamini, 2011; Idler & Benyamini, 1997; Jylhä, 2009). Empirically, SRH has been shown to predict subsequent mortality independent of diagnosed conditions and clinical risk factors, and to correlate with objective indicators such as chronic disease burden, functional limitations, disability progression, and pain-related impairment (DeSalvo et al., 2006; Nielsen et al., 2009; Vogelsang, 2014). In the context of short-run labor-market change, SRH is especially appropriate because early health deterioration often presents first as fatigue, stress, sleep disturbance, or reduced day-to-day functioning—changes that may not yet generate a formal diagnosis but can still meaningfully affect work capacity, attendance, and the ability to sustain employment over the next year (DeSalvo et al., 2006; Jylhä, 2009). Consistent with this logic, research on working conditions routinely employs self-rated or closely related self-reported health outcomes to assess the health consequences of job characteristics (Artazcoz et al., 2005; Burgard et al., 2009; Donnelly, 2022; Erhel et al., 2024; Haile, 2023). At the same time, because CPS does not include objective biomarkers or clinical measures, SRH in this study should be interpreted as perceived health change rather than diagnosed disease progression.

The dependent variable is a three-category measure of between-year change in SRH. For each respondent, we compare SRH at time *t* (“Year 1”) with SRH at time *t*+1 (“Year 2”) across the five CPS categories: excellent, very good, good, fair, and poor. Based on this between-year comparison, we then collapse these transitions into three outcomes: deteriorated (a decline of at least one category), stable (no change), or improved (an increase of at least one category). For instance, a respondent is coded as deteriorated if Year-2 SRH is at least one category worse than Year-1 SRH (e.g., excellent to good, or good to fair); improved if Year-2 SRH is at least one category better than Year-1 SRH (e.g., fair to very good, or poor to fair); and stable if there is no change in SRH between the two interviews (e.g., good to good). This approach follows prior longitudinal work that explicitly models change in SRH as movement between discrete states—improved, stable, or deteriorated—rather than treating SRH as a static level, and demonstrates that such transitions or trajectories are prognostically meaningful for subsequent risk and mortality (Diehr & Patrick, 2001; Nielsen et al., 2009; Sacker et al., 2007; Vogelsang, 2014). This three-category transition measure serves as the dependent variable in the baseline multinomial models in Section 4.1, estimated on the sample of workers continuously employed across the two survey waves.

#### 3.2.2 Expanded Dependent Variable: Health Transition Including Health-Related Work Limitation

Following standard practice in the healthy-worker selection effect (HWSE) literature, respondents who were no longer actively employed by Year 2—because they reduced hours, were not at work, or left the labor force—were excluded from the core analytic sample and therefore dropped from those models. These censoring risks understating health consequences if deteriorating health itself pushes some workers, especially women, out of employment (Arrighi & Hertz-Picciotto, 1994; Buckley et al., 2015; Lea et al., 1999). Prior research shows that health shocks and work-related strain can lead to withdrawal from work or extended absence, and that this process operates differently for men and women (Boden & Galizzi, 2003; Macpherson et al., 2019; Timp et al., 2024). In other words, if unstable work time is associated with health harm that removes people from regular employment, that harm will be largely invisible in a design that follows only those still working.

To address this limitation and complement the baseline dependent variable, we define health-related work limitation (HRWL; hereafter “health limitation”) to capture transitions into labor-force inactivity explicitly attributed to health or medical reasons by Year 2—including working part-time for health reasons, being absent from work due to illness or medical problems, or being out of the labor force because of illness or disability. Here is a detailed variable construction description.

• **Working part-time for health reasons.** We classify respondents as experiencing health-related work limitation if, in Year 2, they report “health/medical limitations” as the main reason for working part-time on CPS variable PEHRRSN1. In the original questionnaire, PEHRRSN1 asks: “What is your main reason for working part-time?” Response options include:
  ○ slack work/business conditions,
  ○ could only find part-time work,
  ○ seasonal work,
  ○ childcare problems,
  ○ other family/personal obligations,
  ○ health/medical limitations,
  ○ school/training,
  ○ retired/social security limit on earnings,
  ○ full-time workweek is less than 35 h, and
  ○ other specified reasons.

Respondents with PEHRRSN1 = “health/medical limitations” are coded into the HRWL component working part-time for health reasons.

• **Being absent from work due to health.** We classify respondents as experiencing health-related work absence if, in Year 2, they are coded as employed but absent from work and report “own illness/injury/medical problems” as the main reason for absence on CPS variable PEABSRSN. In the original CPS questionnaire, this item is asked of all respondents in the “employed-absent” category through the question: “What is the main reason you were absent from work last week?” Response options include:
  ○ layoff,
  ○ slack work/business conditions,
  ○ waiting for a new job to begin,
  ○ vacation/personal days,
  ○ own illness/injury/medical problems,
  ○ childcare problems,
  ○ other family/personal obligations,
  ○ maternity/paternity leave,
  ○ labor dispute,
  ○ weather-affected job,
  ○ training,
  ○ civic/military duty,
  ○ does not work in the business, and
  ○ other specified reasons.

Respondents selecting “own illness/injury/medical problems” are coded into the HRWL component as being absent from work due to health.

• **Being out of the labor force because of illness or disability.** We classify respondents as experiencing health-related labor-force exit if, in Year 2, they are not in the labor force and report “ill-health, physical disability” as their main reason for not looking for work on CPS variable PEDWRSN. Because all respondents in the analytic sample are active labor-market participants in Year 1, this category can also be understood as exiting the labor market for health reasons. In the CPS, respondents are classified as not in the labor force if they were not employed during the reference week and had neither actively searched for work nor been on temporary layoff in the previous four weeks; accordingly, they do not meet the criteria for either employment or unemployment. Among these respondents, PEDWRSN asks: “What is the main reason you were not looking for work during the last 4 weeks?” Response options include:
  o believing no work is available in one's area of expertise,
  o inability to find work,
  o lack of schooling or training,
  o age-related employer discrimination,
  o other discrimination,
  o inability to arrange childcare,
  o family responsibilities,
  o being in school or training,
  o ill-health/physical disability,
  o transportation problems, and
  o other specified reasons.

Respondents with PEDWRSN = “ill-health, physical disability” are coded into the HRWL component being out of the labor force because of illness or disability.

Because all Year 1 respondents in the analytic sample are actively employed (i.e., do not choose employment status due to medical limitations), a transition into HRWL signals the onset of a work-limiting health condition substantial enough to disrupt regular employment, representing a qualitatively more severe form of deterioration than a shift within the self-rated health continuum^32^. This treatment of health-related work limitation as an adverse health transition mirrors prior clinical and occupational research that interprets loss of work capacity or exit from employment after a health event as evidence of meaningful health deterioration (Hassett et al., 2009; Warraich et al., 2018). We therefore treat health limitation as an additional transition category and extend the outcome to four categorical transition states— Improved, Stable, Deteriorated, and Health Limitation—indexed as $Y_{i}\in \{1,0,-1,-2\;\}$. These numeric codes denote category membership only and do not imply equal spacing or a linear health scale.

#### 3.2.3 Independent Variable: Between-Month Work Hour Volatility (Baseline Year)

The independent variable, “work-hour volatility,” is a dispersion measure capturing within-person, between-month variation in usual weekly hours during the baseline year (ASEC “Year 1”). Following Cai's approach (Cai, 2024a, 2024b; Cai & Mattingly, 2025; Cai & Peck, 2022), we compute volatility for each respondent as the standard deviation of month-to-month arc percent changes in hours across the CPS Basic interviews observed in the baseline year. In our approach, the volatility index for person *i* is computed across adjacent CPS months *m* and *m*−1 in Year 1,

$$\text{Arc}\;\text{Change}\;\Delta_{I}=\frac{{Workhours}_{im}-{Workhours}_{im-1}}{\left({Workhours}_{im}+{Workhours}_{im-1}\right)/2\;}$$

$$\text{Volatility}\;\text{Index}\;v_{i}=\sqrt{var\left(\Delta_{I}\right)}$$

Using the pair-specific midpoint in the denominator yields a symmetric percent change—so increases and decreases are on the same scale—and reduces sensitivity to the overall level of hours. Consequently, a volatility value of 0 indicates complete stability (no month-to-month change in usual weekly hours; e.g., working 40 h per week for four consecutive months). In contrast, a volatility value of 1 means that the sample standard deviation of adjacent-month arc changes equals 1. For example, a sequence such as working 20, 60, 60, 20 h for four months, respectively, generates arc changes of 1, 0, and −1, yielding a sample variance $var\left(\Delta_{I}\right)=\frac{{\left(1-0\right)}^{2}+{\left(0-0\right)}^{2}+{\left(-1-0\right)}^{2}}{3-1}=\frac{2}{2}=1$ and a volatility $v_{i}=\sqrt{var\left(1\right)}=1$ —signaling extreme instability in work time. Because the index is a within-person, across-time standard deviation, we require at least three Basic-month interviews in the baseline year to ensure enough adjacent pairs to capture variability while producing a stable estimate. As a robustness check, we also compute volatility as the coefficient of variation of usual weekly hours over the same months (Gennetian et al., 2015); results are substantively unchanged across measures.

#### 3.2.4 Grouping Variable: Gender (Biological Sex)

Prior literature has extensively documented gender and sex differences in SRH reporting (Read & Gorman, 2010; Woo & Zajacova, 2017), in morbidity, disability, and longevity (Krieger, 2003; Short & Zacher, 2022), in exposure to adverse and precarious work conditions (Biswas et al., 2021 & 2022; Campos-Serna et al., 2013), and in labor-market responses after health hazards/injuries (Boden & Galizzi, 2003; Macpherson et al., 2019; Timp et al., 2024). Accordingly, we estimate all models separately for men and women. This grouping strategy provides a clearer picture of sex- and gender-related differences in both the exposure (work-hour volatility) and the outcome (between-year SRH transition). The CPS records biological sex (male/female); we therefore define strata using this measure but interpret observed differences as products of gendered social processes (exposure, vulnerability, and selection). As a robustness check, we also fit pooled models with sex × work-hour-volatility interactions and conduct cross-sex equality tests; results are consistent with the stratified estimates.

#### 3.2.5 Covariates

The key confounder is baseline self-rated health (SRH) measured at ASEC Year 1. Including the full five-category SRH at baseline is a distinct advantage of the linked CPS–ASEC design: unlike repeated cross-sections, the ASEC(t)→ASEC (t+1) linkage allows us to adjust for pre-existing health measured before the volatility window and before the Year-2 outcome. All other controls—and the key independent variable, work-hour volatility, which is constructed from the baseline-year CPS Basic months—are likewise measured prior to the health-transition outcome. This temporal ordering helps reduce (but does not eliminate) concerns about reverse causation (e.g., declining health prompting schedule changes) and strengthens the interpretation of coefficients as associations conditional on prior health and characteristics. That said, the study remains observational; without random assignment and with potential unobserved confounding, the estimates should not be interpreted as causal.

The Year-1 controls capture demographics, human capital, and job context: age, marital status, race/ethnicity, educational attainment, number of children and age of the youngest child in the household, weekly work hours, and major industry^33^ and occupation^34^. All of them are measured on the ASEC month in Year 1. Because the main models are stratified by sex, sex is not entered as a covariate. In light of extensive literature on the COVID period effect (e.g., Bonney et al., 2022;Godefroy & Lewis, 2022; Kniffin et al., 2021; Ravenelle & Kowalski, 2023), we also include period indicators to control the pandemic public health shocks: pre-pandemic (2016–2017, 2017–2018), pandemic (2020–2021, 2021–2022), and post-pandemic (2022–2023, 2023–2024).^35^ The CPS-ASEC dataset also includes other variables available that may be suitable as control variables, such as total family payments for health insurance premiums^36^ and total family out-of-pocket medical payments^37^, though they are not suitable for the current modeling. Appendix B, Appendix Table 1 provides weighted descriptive statistics of all variables currently used in the study.

### 3.3 Analytical Approach

We analyze between-year health transition—coded as ordered categories ranging from deteriorated (−1), stable (0), to improved (+1)—as a function of baseline-year work-hour volatility and Year-1 covariates, estimating all models separately for women and for men. The primary estimator is a multinomial logit model with health transition on “stable (0)” as the reference category, hereafter referred to as a multinomial transition model. In this framework, individuals are classified according to the discrete state they transition into over a fixed observation window, and the model estimates how baseline conditions are associated with the relative likelihood of each mutually exclusive transition outcome. Although multinomial transition models are often applied to multi-stage or sequential processes, a single-transition formulation is appropriate when change can be represented as a one-step movement between states observed at two time points (Breen & Jonsson, 2000; Buis, 2017; Hauser & Andrew, 2006; Karlson, 2011). More broadly, this transition-based perspective is common in public-health and epidemiologic research, where health is often analyzed as movement over time, to characterize health dynamics and subsequent risk (e.g., Nielsen et al., 2009; Sacker et al., 2007; Vogelsang, 2014).

Multinomial transition models are therefore well suited to this study because they align naturally with the panel data structure, the timing of the key predictor, and the measurement of the outcome as a discrete health transition. First, the linked CPS data provide two observations per respondent, defining a single, well-bounded transition window from Year 1 to Year 2 rather than a longer panel suitable for modeling trajectories or cumulative exposure. Second, work-hour volatility is measured at baseline (Year 1), prior to the realization of the health outcome, which allows baseline conditions to be related to subsequent transition probabilities without conflating exposure and outcome within the same period. Third, the outcome of interest is not a continuous change in health level but a categorical transition—deterioration, stability, or improvement—representing mutually exclusive states over this interval. Conceptualizing health change in this way follows prior public-health research that models self-rated health as movement between discrete states over time, rather than as a continuous difference score (Diehr & Patrick, 2001; Nielsen et al., 2009; Sacker et al., 2007; Vogelsang, 2014). All these features define a one-step transition process, for which multinomial transition models provide a direct and appropriate framework by estimating the relative likelihood of each distinct transition outcome within a single observation window.

#### 3.3.1 Baseline Multinomial Transition Model (Employed-Only Sample)

The first set of models—what we refer to as the baseline models and present in Section 4.1—use the analytic sample of continuously employed workers (*N* = 55,062; see Section 3.1). The dependent variable in these models is the three-category SRH transition defined in Section 3.2.1: deteriorated (−1), stable (0), or improved (+1) between ASEC Year 1 and Year 2. Individuals who, by Year 2, had reduced work, were not at work, or left the labor force are excluded from this sample. Accordingly, these models estimate health transitions conditional on remaining continuously employed through Year 2. This design parallels standard practice in the healthy worker survivor effect (HWSE) literature, in which only those who remain in the labor force continue to be observed, and those who step out are no longer observed in the outcome (Arrighi & Hertz-Picciotto, 1994; Buckley et al., 2015; Lea et al., 1999).

Formally, for each sex, we estimate the multinomial logit:

Equation 1 $$\ln\left[\frac{P\left(Y_{i}=k\right)}{P\left(Y_{i}=0\right)}\right]=\alpha_{k}+\beta_{k}*v_{1i}+\gamma_{k}*h_{i1}+\delta_{k}{Χ}_{i1},k\in \left\{-1,+1\right\}$$

where $Y_{i}$ denotes the health transition category of individual i between ASEC Year 1 and Year 2, with $Y_{i}=0$ (stable) serving as the reference outcome. The model thus estimates the relative odds of experiencing deteriorated (−1) or improved (+1) health compared to remaining stable. On the right-hand side, $v_{1i}$ represents baseline-year work-hour volatility; $h_{i1}$ indicates baseline self-rated health (five-category measure); and ${Χ}_{i1}$ is the vector of Year-1 covariates described in Section 3.2.5.

Rather than introducing all controls simultaneously, we ran nested models, gradually incorporating additional covariates to assess robustness. The first specification includes volatility, baseline five-category SRH, age, marital status, race/ethnicity, number of children and age of the youngest child in the household, educational attainment, industry, occupation, and period indicators. The second specification additionally includes weekly work hours as a key control variable, while the final set further adds the interaction between work-hour volatility and weekly work hours. Coefficients are reported as relative risk ratios ("RRRs" in Stata's terminology), representing the multiplicative change in the relative risk of health deterioration or improvement associated with a one-unit increase in volatility, conditional on all Year-1 covariates within the continuously employed sample. All specifications apply the ASEC Year-2 person weights (asecwt_2), which adjust for the ASEC sample design, oversampling, unequal selection probabilities, and nonresponse, ensuring that the analytic sample accurately represents the active labor force that remains observed in Year 2. By construction, these models do not capture health deterioration that coincides with reduced or exited employment, a limitation addressed in the expanded specification below.

#### 3.3.2 Expanded Multinomial Transition Model with Health-Related Work Limitation

Whereas the baseline models estimate health transitions conditional on remaining continuously employed through Year 2, the expanded models target health transitions among the population initially employed at Year 1, regardless of subsequent employment status. Under the four-category new outcome variable that includes HRWL (see Section 3.2.2), we reintroduce 6069 individuals who were excluded from the baseline sample precisely because their health deterioration coincided with health-related reductions in or withdrawal from work, thereby reducing employment-based truncation. Using this larger, less truncated analytical sample (*N* = 61,131), we re-estimate the multinomial logit model in Equation (1) separately for each sex, now with k∈{−2,−1,+1}, so that the model jointly estimates the relative risks of the following versus remaining stable: entering health-related work limitation (−2); experiencing SRH deterioration (−1); and experiencing SRH improvement (+1). All other model settings are the same as in the baseline models.

In the expanded specification, health-related work limitation is modeled as a health-relevant transition state within the multinomial outcome, rather than as a censoring event or a labor-market outcome per se. By design, this second set of models expands both the outcome and the sample, shifting implicit censoring to explicit modeling of health-related exit. Although this approach does not correct selection bias causally, it can reduce mechanical censoring by treating health-driven reductions in or withdrawal from work as adverse health transitions, thereby broadening the empirical scope beyond the continuously employed.

## Footnotes

1 “Just-in-time scheduling” refers to employer practices that adjust workers' hours and shifts on short notice to match real-time business demand. These systems—often enabled by scheduling software—aim to minimize labor costs by staffing only when needed and paying for needed staff and hours only, but they leave workers with unpredictable hours and shifts, unstable incomes, and difficulties managing family or personal responsibilities.

2 On-call arrangements require workers to stay available during set windows and report if summoned; standby time is typically unpaid, and shifts may be confirmed or canceled at short notice, creating income and scheduling uncertainty.

3 “Short-notice schedule changes or cancellations” refer to employer-initiated adjustments to an employee's planned work hours or shifts—such as added, reduced, or canceled shifts—communicated only a few hours or days in advance, often within the same workweek. As of July 2026, U.S. federal labor law generally does not require employers to compensate workers for canceled or reduced shifts, making such short-notice changes particularly undesirable for employees.

4 “Clopening” refers to scheduling an employee to work a late closing shift followed by the next morning's opening shift, often with only a few hours in between (e.g., off at 11:00 p.m., back at 6:00 a.m.). The short turnaround can lead to sleep loss and safety risks, and it complicates commuting, caregiving, and other responsibilities. Some employers or jurisdictions set minimum “rest” intervals or pay premiums for such short-rest shifts, but practices vary widely.

5 The year pair 2018-2019 is not selected to allow a bigger gap from our pre-COVID and mid-COVID samples.

6 We skipped the year pair of 2019-2020, because March 2020 is the very beginning of the COVID-19 pandemic and lockdown, and it's hard to fit this pair to our period categorization.

7 Our independent variable captures actual / realized month-to-month instability in work hours, not workers' prior expectations, advance notice, or schedule control. We therefore treat it as a measure of actual hour instability rather than unpredictability per se, while recognizing that instability and unpredictability often overlap in precarious and low-wage service work (Ananat et al., 2022; Schneider & Harknett, 2019).

8 Because $v_{i}$ is a standard deviation, we require at least three CPS Basic Monthly interviews (yielding at least two adjacent pairs) to produce a stable estimate.

9 The term “arc” comes from geometry and is commonly used in economics to describe change along the segment between two distinct points, rather than change at a single point. Here, the two points are a worker's usual weekly hours in month *m-1* to month *m*. The adjacent-month work-hour changes measured in this study are therefore referred to as “arc changes.”

10 As a robustness check, we also compute volatility as the coefficient of variation of usual weekly hours (Gennetian et al., 2015), with substantively unchanged results.

11 According to the U.S. Bureau of Labor Statistics (BLS), part-time workers are those who usually work fewer than 35 h per week. When a worker reported the reason for part-time schedule is explicitly due to their health or medical conditions (rather than economic or family obligations), it aligns with the concept of “working part-time for health.”

12 In the CPS, absence, or “with a job, not at work,” refers to employed persons who were absent from their jobs for the entire reference week for temporary reasons. When the reported reason for the absence is the respondent's own illness, injury, or medical condition (with or without pay), the case is classified as absent from work due to health.

13 According to the BLS definition, persons not in the labor force are neither employed nor unemployed—that is, they did not work during the reference week and did not actively look for work in the prior four weeks (and were not on temporary layoff). The CPS also records the main reason for nonparticipation. When respondents cite their own illness or disability as the reason, they are classified as "not in the labor force due to health.”

14 As robustness checks, we also fit pooled models with sex × volatility interactions and conduct cross-sex equality tests; conclusions are consistent with the stratified estimates.

15 Shift work, weekend work, and night work are not included as controls because they are conceptually distinct from the focal exposure—month-to-month instability in work hours— and more importantly, are not available across the CPS-ASEC/CPS Basic records used in this study.

16 By “process,” we refer to features such as whether employees are invited for schedule inputs, why work hours fluctuate, and how much advance notice workers receive about schedule changes. These dimensions are conceptually important but are not available in our current dataset. Instead, they are typically asked only in specialized or infrequent survey modules (e.g., the 2017–2018 American Time Use Survey -- Leave module, or 2002, 2006, 2010 and 2014 waves of the General Social Survey Quality of Work Life module) or in data sources not representative for the entire labor force (e.g., the SHIFT project). As a result, the present measure captures the quantity of instability in work hours, rather than the scheduling process that produces it.

17 Fair workweek or secure scheduling regulations have been adopted in several U.S. jurisdictions, including Seattle, Emeryville, California, New York City, Oregon, and Philadelphia. Although provisions vary, these laws generally require advance notice of schedules, predictability pay for employer-initiated short-notice changes, and protections against short-turnaround shifts.

18 The Schedules That Work Act is a proposed federal bill that would give employees the right to request more predictable and stable schedules, and would require covered employers to provide advance schedule notice and compensation for certain short-notice changes (DeLauro, 2019; Miller, 2014). This bill has been introduced in Congress but has not been enacted.
